# Medication and falls in elderly outpatients: an epidemiological study from a German Pharmacovigilance Network

**DOI:** 10.1186/2193-1801-3-483

**Published:** 2014-08-29

**Authors:** Kirsten Heckenbach, Thomas Ostermann, Friedemann Schad, Matthias Kröz, Harald Matthes

**Affiliations:** Havelhoehe Research Institute, Kladower Damm 221, 14089 Berlin, Germany; Institute of Integrated medicine, University of Witten/Herdecke, Gerhard-Kienle-Weg 4, D-58313 Herdecke, Germany

**Keywords:** Fall risk increasing drugs, Fall-related chronic diseases, Falls in elderly

## Abstract

The aim of this study was to investigate the relationship between fall risk increasing drugs (FRIDS) and the risk of falls in regard to fall-related chronic diseases. In total, 39 primary care physicians in Germany participated in the EvaMed Pharmacovigilance Network. Antihypertensives, non-steroidal anti-inflammatory drugs, hypnotics and sedatives, antidepressants and psycholeptics were labelled as *FRIDS*. A *fall* was defined according to a diagnosis in the chapter *Injury or poisoning* (S00-T14 in International Statistical Classification of Diseases 10th Revision (ICD-10)). Patients older than or equal to 65 years with at least two doctor’s visits were included. FRIDS were prescribed for 1768 patients from a total of 5124 patients included in the analysis. FRIDS and seven chronic diseases were statistically significant associated with a higher risk of experiencing a fall. The risk was highest for patients with a diagnosis abnormalities of gait and mobility, vertigo, visual -impairment and weight loss, and increased by 50-90% with arthritis, diseases of arteries, arterioles and capillaries and heart failure. From patients (N = 425) with at least one diagnosis of fall, 219 patients were prescribed FRIDS. In 100 (45.7%) of cases the diagnoses for fall were made before and in 105 (47.9%) of cases at least a month after the prescription of FRIDS. 14 (6.4%) patients had a prescription of FRIDS and a diagnosis of fall within one month. Perceptual disorders, low walking speed and pain are prominent predictors for falls in the elderly. A prescription of FRIDS selects more vulnerable patients having a higher risk of falls. However, experiencing a fall is mainly due to the disease followed by treatment. Thus, not prescribing FRIDS will avoid only a small number of falls.

## Introduction

Enabling healthy human aging is one of the most challenging tasks for health care systems around the world. At present the percentages of older people in the populations of European countries are steadily increasing, leading to heightened requirements for socio-medical services and health care. In 2011 Germany counted for a total of 2.5 million care-dependent elderly persons, which was an increase of 10% compared to the year 2007 (Statistisches Bundesamt, Wiesbaden 2013). Apart from logistical problems such as the process of admitting elderly persons to a nursing home (Tabali et al. 2013), the prescribing of medication to elderly patients becomes an increasing medical, but also economical issue. The medication of elderly patients is a risk assessment between wanted and adverse drug reactions. In particular the intake of multiple medications leads to a lower medication adherence (Hubbard et al. 2013), failing vision and impaired physical functioning in elderly patients (Scheffer et al. 2013). According to Tinetti (2003) the intake of four or more prescribed remedies denotes an established risk factor for falls in older people.

Today a wide range of evidence exists regarding the association of polypharmacia and the risk of falls. According to a meta analysis by Woolcott et al. (2009), the medication groups of psycholeptics, psychoanaleptics, non-steroidal anti-inflammatory drugs and antihypertensives drugs increase the risk of falls. The influence of fall risk increasing drugs (FRIDS) on falls or fall-related injuries of elderly persons has been examined in a variety of studies including hip fractures (Guo et al. 1998), or focused on therapeutic groups such as psychotropic agents (Pierfitte et al. 2001; Thapa et al. 1995, 1998; Ziere et al. 2008; Milos et al. 2014). Other studies have criticized these results and state that fall-related injuries could also be caused by morbidity (Bauer et al. 2012). In addition extensive research was conducted into the field of disabilities and fall-related chronic diseases (Dunn et al. 1992; Gabbe et al. 2013; Haagsma et al. 2011; Pilotto et al. 2012; Stenhagen et al. 2013).

Up to 2013 only two studies have investigated the effects of FRIDs using data within Germany (Bauer et al. 2012; Schwab et al. 2000). In a case control study, Schwab et al. (2000) found the intake of benzodiazepines to be a prominent risk-factor for falls and hip fractures in the elderly. The second study of Bauer et al. (Bauer et al. 2012) used administrative data from a German health insurance company to show that incidence of injuries strongly increases with doses of antidepressants, anxiolytics, hypnotics, sedatives, and antiarrhythmics. While the first study mainly concentrated on falls related to a specific class of drugs, the second study chose a more general approach. Our study is the first of its kind investigating FRIDS medication with regard to fall-related chronic diseases. The aim of the current study was to investigate the relationship between FRIDS and the risk of falls with regard to fall-related chronic diseases in patients within a network of German general practitioners.

## Material and methods

### Design

In total, 39 primary care physicians in Germany participated in a multicenter observational study from 2004 to 2010. All of the participants were members of the EvaMed Pharmacovigilance Network, which aims to evaluate complementary remedies in usual care with regard to prescribing patterns, efficacy, and safety (Jeschke et al. 2009a, [b]). During the study participating physicians continued to follow their routine documentation procedures, recording all treatment relevant diagnoses and all prescriptions for each consecutive patient using their existing computerized patient documentation system. Data were transferred to the EvaMed Documentation server including an exact time stamp. Diagnoses were coded according to the 10th revision of the International Classification of Diseases (ICD-10). Prescribed drugs were documented using the German National Drug Code (German: Pharmazentralnummer; PZN) and categorized by the anatomical, therapeutic chemical (ATC) classification system. Missing ICD-10 and PZN codes were added by the study center. Data were included in the analysis if patients were at least 65 years old, or turned 65 during the study period, and visited the physician at least twice during the study period, since two time events were included in the analysis which was based on a diagnosis of falls in relation to a prescription of FRIDS.

### Definitions: FRIDS, (Recurrent) falls and fall related chronic diseases

According to a meta-analysis antihypertensives (C02), non-steroidal anti-inflammatory drugs (NSAIDs) (M01A), hypnotics and sedatives (N05C), antidepressants (N06A), psycholeptics and psychoanaleptics combination (N06C) were named as *FRIDS* (Woolcott et al. 2009). Diagnoses coded by ICD-10 as injury or poisoning (S00 - T14) were regarded as *falls*. Excluded were diagnoses relating to bites from animals, injuries caused by knives or burns and sequelae of injuries (war). A fall was defined as recurrent falls, if different ICD-10 codes were recorded or the same ICD-10 code was recorded after three quarters of a year (274 days), to avoid counting duplicated events with long lasting recovery. A possible connection between the intake of FRIDS and a diagnosis of fall was assumed if the diagnosis was made within one month after the prescription, with the consideration that the patient would be prescribed FRIDs for an acute disease.

Fall-related chronic diseases were categorized in abnormalities of gait and mobility (incl. Parkinson disease) (ICD-10: R26, R27,G20-G26)) (Stenhagen et al. 2013) arthritis (ICD-10: M05, M06, M13, M15- M19, M47, M48) (Stenhagen et al. 2013; Tinetti and Kumar 2010; Oliver et al. 2004; Deandrea et al. 2010), cerebrovascular disease (ICD-10: I60-I69)) (Dunn et al. 1992), chronic ischaemic heart disease chronic ischaemic heart disease (ICD-10: I25) I25) (Dunn et al. 1992; Tinetti and Kumar 2010; Oliver et al. 2004; Deandrea et al. 2010), dementia dementia (ICD-10: F00-F05, F10, F20)) (Tinetti and Kumar 2010; Oliver et al. 2004; Deandrea et al. 2010), depression depression (ICD-10: F32, F33)) (Tinetti and Kumar 2010; Oliver et al. 2004; Deandrea et al. 2010) diabetes (ICD-10: E10- E14, O24.0-O24.4, R73)) (Tinetti and Kumar 2010; Oliver et al. 2004; Deandrea et al. 2010), diseases of arteries, arterioles and capillaries diseases of arteries, arterioles and capillaries (ICD-10: I70-I79, I80-I89, I95-I99)) (Dunn et al. 1992; Tinetti and Kumar 2010; Oliver et al. 2004; Deandrea et al. 2010), hearing impairment hearing impairment (ICD-10: H90, H91)) (Dunn et al. 1992), heart failure (ICD-10: I50, I09, I11)) (Dunn et al. 1992; Stenhagen et al. 2013; Tinetti and Kumar 2010; Oliver et al. 2004; Deandrea et al. 2010), hypertension (ICD-10: I10) I10) (Dunn et al. 1992), intervertebral disc disorders, dorsalgia (ICD-10: M51, M53, M54) (Tinetti and Kumar 2010; Oliver et al. 2004; Deandrea et al. 2010; Anonymous 2001; Rubenstein 2006) malignant neoplasm (ICD-10: C00-C97) C00-C97) (Dunn et al. 1992) osteoporosis (ICD-10: M80) (Dunn et al. 1992), sleep disorders (ICD-10: G47), vertigo (ICD-10: H81, H82, R42) H81, H82, R42) (Stenhagen et al. 2013), visual impairment visual impairment (ICD-10: H53-H54)) (Dunn et al. 1992), weight loss weight loss (ICD-10: E41, E46, E64, R63.4)) (Dunn et al. 1992), incontinence (ICD-10: R32 or ATC code = V07AN) R32 or ATC code = V07AN) (Tinetti and Kumar 2010; Oliver et al. 2004; Deandrea et al. 2010) and disability in moving as a pand disability in moving as a pand disability in moving as a prescription of walking and mobility aids.

### Statistics

Descriptive analysis was used to determine prescription rates and the distribution of chronic diseases. Descriptive statistics are presented with 95% confidence intervals (CI).

To identify independently associated risk factors with a diagnosis of fall as the dependent variable, a blockwise modeling strategy was conducted. In a first model, age and sex were used as continuous variables. In a further model, each fall-related chronic disease was tested as a risk factor adjusted for age and sex. A second model tested the association of falls with all fall-related chronic diseases with a p value < 0.15 adjusted for age and sex and an OR >1. In a third model, the association between FRIDS and falls was tested, controlling for age, sex and fall-related chronic diseases Adjusted odds ratios (AOR) with 95% confidence intervals (CI) were calculated. The significance level was set at α = 0.05.

Hosmer-Lemeshow goodness-of-fit test was used to assess how well the chosen model fits the data. Statistical analysis was performed with R: ‘A Language and Environment for Statistical Computing (2012).

The present study was based on secondary data provided by physicians. As such, the recommendations for good practice in secondary data analysis (e.g. anonymization of data on prescriptions and diagnoses) developed by the German Working Group on the Collection and Use of Secondary Data (Ihle et al. 2012) were applied in full. As no experimental research or intervention on patients was applied, no ethical approval was needed. Nevertheless, the study was approved by the responsible data security official.

## Results

Data were provided by 26 from a total of 39 physicians from the EvaMed network. 5,124 patients with a mean age of 73 years (CI 72.7-73.2) were included in the final analysis. Female patients were 1.6 years older than male (73.5 years, CI 73.3-73.8 versus 71.9 years, CI 71.7-72.1). On average patients were under medical attention by the physician for two years. During the study period 9,031 FRIDS were prescribed to 1,768 (100%) patients. NSAIDs were prescribed most frequently, making up 37.6% (n = 3,398) of prescriptions to 1,030 (58.3%) patients, followed by prescriptions for hypnotics and sedatives (29.3%, n = 2646) to 465 (26.3%) patients and antidepressants (23.5%, n = 2,120) to 555 (31.4%) patients. Antihypertensives were only prescribed 867 times (9.6%) to 242 (13.7%) patients. No prescription of combinations of psycholeptics and psychoanaleptics was documented. At least one diagnosis of fall had 425 patients, more than half of them received a prescription of FRIDS (n = 219) (Table 1).

**Table 1:** **Description of patients 65 years and older**

		Falls	No falls	Totals
		Mean (95% CI)	Mean (95% CI)	Mean (95% CI)
Age (years)		74.5(73.7-75.4)	72.8(72.6-73)	73.0(72.7-73.2)
		**n(%)**	**n(%)**	**N(%)**
Gender				
	Male	136(7.5)	1,683(92.5)	1,819(35.5)
	Female	289(8.7)	3,016(91.3)	3,305(64.5)
Outcome				
	Injuries and poisoning^a^	425(100)	4,699(91.7)	425(8.3)
Diagnosis				
	Recurrent injuries or poisoning >1	79(100)	5,042(98.5)	79(1.5)
	Number of fall ascociated chronic diseases <=1	138(5.7)	2,275(94.3)	2,413(47.1)
	Chronic diseases >=2	287(10.6)	2,424(89.4)	2,711(52.9)
	Abnormalities of gait and mobility (incl. parkinson)^b^	24(19)	102(81)	126(2.5)
	Dementia^f^	36(15.3)	199(84.7)	235(4.6)
	Depression^g^	82(13.6)	522(86.4)	604(11.8)
	Diabetes^h^	68(10.4)	589(89.6)	657(12.8)
	Diseases of arteries, arterioles and capillaries^i^	129(13.2)	851(86.8)	980(19.1)
	Hearing impaiment^j^	21(21.4)	77(78.6)	98(1.9)
	Heart failure^k^	80(16.2)	413(83.8)	493(9.6)
	Hypertension^l^	186(9.6)	1,754(90.4)	1,940(37.9)
	Intervertebral disc disorders, dorsalgia^m^	105(12.5)	735(87.5)	840(16.4)
	Malignant neoplasm^n^	66(5.7)	1,088(94.3)	1,154(22.5)
	Osteoporose^o^	3(8.3)	33(91.7)	36(0.7)
	Sleep disorders^p^	55(15)	311(85)	366(7.1)
	Vertigo^q^	38(25.5)	111(74.5)	149(2.9)
	Visual impairment^r^	19(22.1)	67(77.9)	86(1.7)
	Weight loss^s^	7(24.1)	22(75.9)	29(0.6)
	Incontinence^t^	60(15.2)	336(84.8)	396(7.7)
	Disability in moving^u^	27(10.3)	234(89.7)	261(5.1)
Medication				
	Fall risk increasing drugs (FRIDS)^v^	219(12.4)	1,549(87.6)	1,768(34.5)

References and ICD10 Codes: a) Injuries and poisoning (S00-S99, T00- T14), b) abnormalities of gait and mobility (incl. Parkinson) (ICD-10: R26, R27,G20-G26) (Stenhagen et al 2013), c) arthritis (ICD-10: M05, M06, M13, M15- M19, M47, M48) (Stenhagen et al. 2013; Tinetti and Kumar 2010; Oliver et al. 2004; Deandrea et al. 2010), d) cerebrovascular disease (ICD-10: I60-I69) (Dunn et al. 1992), e) chronic ischaemic heart disease (ICD-10: I25) (Dunn et al. 1992; Tinetti and Kumar 2010; Oliver et al. 2004; Deandrea et al. 2010), f) dementia (ICD-10: F00-F05, F10, F20) (Tinetti and Kumar 2010; Oliver et al. 2004; Deandrea et al. 2010), g) depression (ICD-10: F32, F33) (Tinetti and Kumar 2010; Oliver et al. 2004; Deandrea et al. 2010), h) diabetes (ICD-10: E10- E14, O24.0-O24.4, R73) (Tinetti and Kumar 2010; Oliver et al. 2004; Deandrea et al. 2010), i) diseases of arteries, arterioles and capillaries (ICD-10: I70-I79, I80-I89, I95-I99) (Dunn et al. 1992; Tinetti and Kumar 2010; Oliver et al. 2004; Deandrea et al. 2010), j) hearing impairment (ICD-10: H90, H91) (Dunn et al. 1992), k) heart failure (ICD-10: I50, I09, I11) (Dunn et al. 1992; Stenhagen et al. 2013; Tinetti and Kumar 2010; Oliver et al. 2004; Deandrea et al. 2010), l) hypertension (ICD-10: I10) (Dunn et al. 1992), m) intervertebral disc disorders, dorsalgia (ICD-10: M51, M53, M54) (Tinetti and Kumar 2010; Oliver et al. 2004; Deandrea et al. 2010; Anonymous 2001; Rubenstein 2006], n) malignant neoplasm (ICD-10: C00-C97) (Dunn et al. 1992), o) osteoporosis (ICD-10: M80) (Dunn et al. 1992), p) sleep disorders (ICD-10: G47), q) vertigo (ICD-10: H81, H82, R42) (Stenhagen et al. 2013), r) visual impairment (ICD-10: H53-H54) (Dunn et al. 1992), s) weight loss (ICD-10: E41, E46, E64, R63.4) (Dunn et al. 1992), t) incontinence (ICD-10: R32 or ATC code = V07AN) (Tinetti and Kumar 2010; Oliver et al. 2004; Deandrea et al. 2010), u) disability in moving as a prescription of walking and mobility aids, v) Fall risk increasing drugs (FRIDS) (ICD-10: C02, M01A, N05C, N06A, N06C) (Woolcott et al. 2009).

In 14 of these cases (6.4%), a first diagnosis of fall occurred within one month after the first prescription of FRIDS. In 100 (45.7%) of cases the diagnoses for fall were made before (at mean 231.8 (CI 144.2-319.4) days) and in 105 (47.9%) of cases at least a month (at mean 768.6 (CI 625.5-911.7) days) after the prescription of FRIDS.

From the 14 patients with a possible causative relationship between prescription of FRIDS and falls, patients were prescribed seven times NSAIDs, three times hypnotics and sedatives and antidepressants, and one prescription of antipsychotics (Table 2). The diagnoses were fractures (9), contusions (5) and superficial injuries (4). Body parts included head (1), upper limb (6), trunk (5), lower limb (4) and twice parts were not specified. Apart from two cases of fractures of the spine returning to the physician two days after the prescription of FRIDS, it is likely for all other cases that patients initially received the prescription and had fallen afterwards. The injuries were typical for breaking a fall with hands affecting wrist, hands and forearm and without breaking a fall with hands, affecting the shoulder and the trunk (DeGoede et al. 2003). Apart from three patients, patients generally had between 3 to 11 diagnoses of fall-related chronic diseases. Two patients had been falling recurrently with three diagnoses of falls.

**Table 2:** **Single patients with a diagnosis fall within one month after prescription of FRIDS, falls sorted by ascending distance to the prescription of FRIDS**

Patient	ATC code	Name	ICD-10	icd10 text	Days ^a^	Age ^b^
1	M01AE01	Ibuprofen	T08.0	Fracture of spine, level unspecified	2	66
2	N05CC01	Chloral hydrate	T08.0	Fracture of spine, level unspecified	2	89
3	M01AB05	Diclofenac-	S42.3	Fracture of shaft of humerus	5	80
4	N05AH04	Quetiapine	S00.0	Superficial injury of scalp (multi prescriptions 4.fall)	6	74
5	N06AP01	St. John’s wort	S63.50	Sprain and strain of wrist	6	76
6	M01AH05	Etoricoxib	S72.9	Fracture of femur, part unspecified	7	78
7	N06AB04	Citalopram	S42.20	Fracture of upper end of humerus	8	89
8	M01AB05	Diclofenac-	T08.0	Fracture of spine, level unspecified	11	62
9	M01AE17	Dexketoprofen	S42.3	Fracture of shaft of humerus	13	91
			S52.50	Fracture of lower end of radius		
			S70.0	Contusion of hip		
10	N05CF01	Zopiclone	S72.00	Fracture of neck of femur	17	66
11	M01AB05	Diclofenac	S50.0	Contusion of elbow	28	93
			S80.0	Contusion of knee		
12	N05CD06	Lormetazepam	S33.5	Sprain and strain of lumbar spine	28	65
13	M01AE01	Ibuprofen	T14.1	Open wound of unspecified body region	29	62
14	N06AP01	St. John’s wort	T11.05	Superficial injury of upper limb, level unspecified	31	76
			T14.00	Superficial injury of unspecified body region		

^a^Days between prescription and fall.

^b^Age at diagnosis in years.

The diagnoses of falls (n = 515) were analysed according to the type and location of the injury. The type of the diagnoses were mainly fractures (36.3%) and superficial injuries (34.0%) (Table 3). Injuries occurred mainly at the upper limb (21%) and the trunk (19%). The lower limb (leg, knee, ankle foot) was stated in 11% followed by hip and thigh (8%). Thirty seven percent of the injuries had multiple locations or were not documented with a specific body region.

**Table 3:** **Types of injury and body region affected**

		n (%)	n
Dislocation, sprain and strain of joints and ligaments		70(13,6)	
	Head		
	Hip and thigh		2
	Lower limb		16
	Trunk		12
	Unspecified body region		29
	Upper limb		11
Fractures		187(36,3)	
	Head		6
	Hip		29
	Lower limb		22
	Multiple and unspecified body region		19
	Trunk		61
	Upper limb		50
Open wound and injuries		79(15,5)	
	Head		5
	Hip and thigh		6
	Lower limb		8
	Multiple and unspecified body region		22
	Trunk		2
	Upper limb		36
Superficial		175(34)	
	Head		7
	Hip and thigh		5
	Lower limb		10
	Multiple and unspecified body region		121
	Trunk		23
	Upper limb		9
Intracranial injury	Head		4
Sum		515(100)	515

We conducted three logistic regression models testing the hypothesis that age and sex, chronic diseases and FRIDS would be independent risk factor for falls. In the first model, increasing age was a statistically significant risk factor for falls. Although sex was not statistically significant associated it remained a strong known confounder in the model. Whilst controlling for age and sex, 10 out of 19 diagnoses were significantly associated with falls while malignant neoplasm was a protective factor in respect to falls (Table 4). The risk doubled at least with the diagnoses of weight loss, vertigo, abnormalities of gait and mobility. The risk increased more than 50% with a diagnoses of arthritis, heart failure, sleep disorder or visual impairment. Patients with a diagnoses of depression, intervertebral disc disorders and diseases of arteries had an up to 50% increased risk. Adding FRIDS to the model with independently associated diagnoses, the prescription of FRIDS had a 70% increased risk. The remaining risk factors in the third model besides FRIDS were diseases not treatable with FRIDS, but associated with balance (vertigo, visual impairment, abnormalities of gait and mobility), gait speed (weight loss and heart diseases) and pain from a degenerative disease (arthritis). Factors statistically significant associated with falls in the model excluding FRIDS (depression, sleep disorders and intervertebral disc disorders), were no longer associated, when FRIDS was included in the model. In the second model, the diagnoses depression, sleep disorders and intervertebral disc disorders act as a representative for the medication or further diagnoses leading to dizziness and low walking speed. In the third model, FRIDS remained as the important risk factor for falls.

**Table 4:** **Regression models with falls as dependent variable**

	Model 1			Model 2		Model 3		Final Model	
	AOR (95% CI)	Crude OR (95% CI)	p value	AOR (95% CI)	p value	AOR (95% CI)	p value	AOR (95% CI)	p value
Age in years	1.024(1.012-1.036)		<0.001	1.01.0(0.997-1.024)	<=0.15	1.011.0(0.998-1.024)	<=0.15	1.013(1.001-1.026)	<0.05
Female gender	1.1(0.9-1.4)		>0.15	1.0(0.8-1.3)	>0.15	1.0(0.8-1.3)	>0.15	1.0(0.8-1.3)	0.675
Abnormalities of gait and mobility (incl. parkinson)		2.6(1.6-4)	<0.001	2.1(1.3-3.3)	<0.05	2.0(1.2-3.2)	<0.05	2.1(1.3-3.4)	<0.05
Arthritis		2.0(1.6-2.5)	<0.001	1.5(1.2-1.9)	<0.001	1.4(1.1-1.8)	<0.05	1.5(1.2-1.9)	<0.001
Cerebovascular disease		1.8(1.3-2.5)	<0.001	1.3(0.9-1.9)	<=0.15	1.4(0.9-1.9)	<0.1	-	-
Chronic ischaemic heart disease		1.1(0.8-1.5)	>0.15	-	-	-	-	-	-
Dementia		1.8(1.2-2.6)	<0.05	1.3(0.8-1.9)	>0.15	-	-	-	-
Depression		2.0(1.5-2.5)	<0.001	1.4(1.1-1.9)	<0.05	1.3(1.0-1.7)	<0.1	-	-
Diabetes		1.3(1.0-1.7)	<0.1	1.1(0.8-1.4)	>0.15	-	-	-	-
Disability in moving		1.1(0.7-1.7)	>0.15	-	-	-	-	-	-
Diseases of arteries, arterioles and capillaries		1.9(1.6-2.4)	<0.001	1.4(1.1-1.8)	<0.05	1.4(1.1-1.8)	<0.05	1.5(1.2-1.9)	<0.001
Hearing impaiment		2.9(1.7-4.7)	<0.001	1.6(0.9-2.8)	<0.1	1.7(1.0-2.8)	<0.1	-	-
Heart failure		2.2(1.6-2.8)	<0.001	1.6(1.2-2.1)	<0.05	1.6(1.2-2.1)	<0.05	1.7(1.3-2.3)	<0.001
Hypertension		1.3(1.0-1.6)	<0.05	1.1.0(0.9-1.4)	>0.15	-	-	-	-
Incontinence		1.9(1.3-2.5)	<0.001	1.3(0.9-1.8)	<=0.15	1.3(0.9-1.8)	<=0.15	-	-
Intervertebral disc disorders, dorsalgia		1.8(1.4-2.3)	<0.001	1.3(1.0-1.7)	<0.05	1.2(0.9-1.6)	<=0.15	-	-
Malignant neoplasm		0.6(0.5-0.8)	<0.001	-	-	-	-	-	-
Osteoporose		0.9(0.2-2.6)	>0.15	-	-	-	-	-	-
Sleep disorders		2.1(1.5-2.8)	<0.001	1.5(1.0-2.0)	<0.05	1.4(1.0-1.9)	<0.1	-	-
Vertigo		3.7(2.5-5.4)	<0.001	2.5(1.6-3.8)	<0.001	2.5(1.6-3.7)	<0.001	2.9(1.9-4.2)	<0.001
Visual impairment		3.4(1.9-5.5)	<0.001	1.9(1.0-3.3)	<0.05	1.9(1.0-3.3)	<0.05	2.1(1.2-3.6)	<0.05
Weight loss		3.6(1.4-8.2)	<0.05	2.9(1.1-6.7)	<0.05	2.8(1.1-6.6)	<0.05	3.1(1.2-7.1)	<0.05
Fall risk increasing drugs (FRIDS)			<0.001	-	-	1.5(1.2-1.9)	<0.001	1.7(1.4-2.1)	<0.001

Regression models with falls as dependent variable: Model 1 age and sex. Each fall-related chronic diseases, was tested as a risk factor adjusted for age and sex. Model 2 included all fall-related chronic diseases with a p value < 0.15 adjusted for age and sex and an OR >1. Model 3, including FRIDS and all fall-related chronic diseases from Model 2 with a p value < 0.15 adjusted for age and sex.

## Discussion

Firstly, our study contributes to the existing literature by providing an analysis of FRIDS under consideration of fall-related chronic diseases for the first time using German data. Secondly, the empirical analysis is based on routine data from physicians with 5,124 patients and their data on prescriptions and diagnoses, including exact dates. In regard to the constitution of the pharmacovigilance network with active documentation of all adverse events the EvaMed database exceeds all other secondary data analysis e.g. German health insurance analysis. This enabled us to analyse the chronological sequence of prescription of FRIDS and possible falls.

Although the routine data do not document the reason for the fall, we assume most of the documented falls are fall-related and not caused by any other external causes like car accidents. A prospective study with all residents (n = 679) aged 75 or 80 years from a city in Finland was conducted to investigate types of injury and body regions affected in injurious falls over a 10-year period. All falls that required medical care were registered. From 718 accident-related injuries 88% were considered fall-related. The most common injuries were fractures of the upper limb and the hip. The frequencies of fractures and superficial injuries are comparable with our distribution. In contrast to the study from Finland, we have only a few injuries requiring first aid (superficial injuries of the head and intracranial injuries) or treatment in a hospital (fracture of the hip).

Our main results were that falls are potentially associated with three factors:

- Firstly, patients with a prescription of FRIDS are more vulnerable and at a higher risk of falls, regardless of a timely connection between drug use and fall. Although the association between the use of FRIDS with falls in elderly individuals has been demonstrated by Woolcott et al. (2009), the connection between drug intake and falls has not been researched very widely in the past. In 2003, Lamb et al. (2003) noticed the lack of evidence with respect to the temporal association between supposed risk factors and falls. Armstrong et al. (2005) also raised the need for prospective studies to *characterise the temporal nature of the observed associations*.
- As a second factor we found that patients with perceptional disorders, visual and balance impairment had the highest risk of experiencing a fall, which is in accordance with a study about recurrent fallers and their visit to an emergency department after a fall (Askari et al. 2013). Besides known FRIDS (analgetics, anti-Parkinson drugs, antipsychotics and antidepressants, drugs for acid-related disorders), nasal preparations and ophthalmologicals were significantly associated with a visit in the emergency department (Askari et al. 2013).
- As a third factor we were able to confirm reduced mobility (Stenhagen et al. 2013), low walking speed (Quach et al. 2011; Muraki et al. 2013) and pain (Muraki et al. 2013; Muraki et al. 2011) as prominent predictors for falls in the elderly. The risk of falling for patients with pain could be regarded as an adverse drug reaction to NSAIDs, providing the chronology is not being taken into account.

O’Neil et al. (2012) summarised studies and meta-analyses on the treatment of pain with a focus on falls as adverse drug reaction. A meta-analysis found that patients exposed to central acting analgesics (opioids) had a 38% increased risk of fractures compared to non-treated controls (Takkouche et al. 2007). Comparing peripheral acting analgesics (NSAID) and opioids in older adults with arthritis revealed a five-fold increased risk of fracture in those using opioids. (Miller et al. 2011). Two studies detected a dose dependence, where the risk of falls increased with higher doses of opioids (Muraki et al. 2013; Saunders et al. 2010). The risk of falling increased also if opioids were used with non-opioid analgesics. One analysis was conducted with regard to gait balance problems (Buckeridge et al. 2010). Whether the falls originated from the analgesics or from the underlying disease remained unanswered. Most studies were unable to control for pain severity which could have had an important confounding effect (O’Neil et al. 2012). Patients that received a prescription for opioids were treated for moderate to severe pain, while NSAID’s were generally used to treat only mild pain (Schwabe and Paffrath 2014).

Only few studies investigated the effect of the withdrawal of medication. Withdrawal significantly reduced the expected rate of falls (incidence density), but not the cumulative falls incidence (Campbell et al. 1999; Zermansky et al. 2006)^.^ In more than 50% of patients from a geriatric outpatient population (n = 139) a reduction of the risk of falling was achieved by the withdrawal of cardiovascular drugs, antihypertensives, antiarrhythmics, nitrates and other vasodilators, digoxin and β-adrenoceptor blocker .

Further correlations between medication and falls were found in other studies. Any type of change in the class of psychotropic drugs increased the risk of a fall (Echt et al. 2013). In addition, the risk of a fall increased independent of the drug class in patients with diabetes if they were prescribed four or more medications (Huang et al. 2010), confirming the results of Tinetti (2003).

A reduction of falls in frail elderly could not be achieved with a single intervention and it is questionable whether the withdrawal of FRIDs medication would have an effect. Even multifactorial interventions recommended by an international guideline (Anonymous 2001) were not supported by the results of studies.

A Health technology assessment (HTA) for the German situation addresses the effectiveness of single interventions and complex programs for the prevention of falls and fall-related injuries. The assessment included 184 out of 12,000 studies. A prior assessment of individuals at risk of falling had no or little effect. Positive effects of exercise interventions in relatively young and healthy seniors were observed, while the opposite was noted in the fragile elderly. For this specific vulnerable population modification of the housing environment showed protective effects. A number of low quality studies and inconsistent results led to the conclusion that the effectiveness of the following interventions is unclear: correction of vision disorders, modification of psychotropic medication, vitamin D and nutritional supplementation, psychological interventions, education of nursing personnel, multiple and multifactorial programs as well as the application of hip protectors (Balzer et al. 2012).

Limitations:

- The present study has important limitations which should be taken into account when interpreting the results.
- Firstly, each diagnosis of poisoning and injury was regarded as a fall. This might overestimate the number of falls.
- Secondly, although physician-prescribing data were subjected to an internal review, coding inaccuracies cannot be ruled out entirely.
- Thirdly, data on additional medication use in patients who visited several physicians simultaneously were unavailable and might underestimate the use of FRIDS.

## Conclusions

In addition to the normal risk of falling, people included in the elderly population lose the ability to prevent falls and the protection reaction to minimize the consequences of falls. Each patient at a higher risk should be screened for their causal reasons and their individual modifiable factors for fall prevention. Main issues are pain reduction, stable walking and adequate medication in regard to the quantity and the duration. Our data suggest not that FRIDS medication are the source of falls but are often applied in risk diseases for falls. For more evidence regarding the temporal correlation of drug intake and falls we suggest to analyse computerized prescription databases to understand the temporal nature of falls in the elderly. Furthermore, the evaluation of the necessity of medication, especially psychotropic substances, should be examined for each individual at least yearly (Bergert et al. 2013).

## Abbreviations

AOR: Adjusted ODDS ratio
ATC: Anatomical, therapeutic chemical
CI: 95% confidence intervals
FRIDS: Fall risk increasing drug
ICD-10: 10th revision of the International Classification of Diseases
NSAID: Non-steroidal anti-inflammatory drugs.
